# Less is more: calorie restriction as a therapeutic for mental health disorders

**DOI:** 10.3389/fpsyt.2025.1584890

**Published:** 2025-06-16

**Authors:** Jason C. D. Nguyen, Antonina Govic, Elizabeth A. Levay, Matthew D. Zelko, Thiruma V. Arumugam, Jim Penman, Terrance G. Johns, Zoran Boskovic

**Affiliations:** ^1^ Epigenes Australia Pty Ltd., Melbourne, VIC, Australia; ^2^ School of Agriculture, Biomedicine and Environment, La Trobe University, Melbourne, VIC, Australia; ^3^ School of Psychology and Public Health, La Trobe University, Melbourne, VIC, Australia

**Keywords:** anxiety, depression, substance use disorders, addiction, calorie restriction, calorie restriction mimetic

## Abstract

Anxiety, depression, and substance use disorders are prevalent mental health disorders that have debilitating health outcomes, and current treatment options are not always efficacious or tolerable. Calorie restriction (CR) has various health benefits, with research efforts focused on its effects in improving metabolic health and delaying biological aging. Recent studies have indicated that CR can also improve anxiety-, depression- and addiction-like symptoms and behavior. Similar benefits have also been observed in studies investigating a range of CR mimetics (CRMs) - molecules that mimic one or more of the physiological effects of CR without dietary restriction - indicating that both CR and CRMs could be used to assist in treating these symptoms. Here, we summarize the current evidence for the potential use of CR and select CRMs in the treatment of anxiety, depression, and addiction, as well as the possible molecular mechanisms underlying these beneficial effects. Finally, we propose novel molecular signatures that could be exploited to screen for novel CRM candidates.

## 1 Introduction

Globally, an estimated one in eight people live with a mental disorder (1). In Australia, the 2020–2022 National Study of Mental Health and Wellbeing reported that 42.9% of individuals aged 16–85 had experienced a mental disorder in their lifetime, 21.5% had a 12-month disorder, and 38.8% of those aged 16–24 had a 12-month disorder (2). Mental disorders - such as anxiety, depression, and substance use disorders - impair cognition, emotional regulation, and behavior. They are leading causes of disability and significant contributors to premature mortality (1, 3). The COVID-19 pandemic served to further exacerbate the mental health crisis, with patients with preexisting psychiatric disorders reporting worsening mental health, and the general public showing lower psychological well-being compared to the pre-COVID era (4). Mental disorders account for an estimated 418 million disability-adjusted life years, with an associated global economic burden of approximately USD 5 trillion (5). While current treatments - including psychotherapy and pharmacological interventions - offer benefits, they are often limited by delayed onset, inadequate efficacy, side effects, or risk of dependence (6, 7). These limitations underscore the urgent need for novel, safe, and effective treatment strategies.

In recent years, a growing body of research has begun to explore the potential of metabolic interventions in addressing the limitations of conventional mental health treatments. Among these, calorie restriction (CR) - a reduction in calorie intake without malnutrition – has emerged as a promising avenue. This dietary regimen has gained significant traction in recent years due to evidence indicating a range of benefits beyond delaying biological aging (8–10) that include improvements in metabolic health (11–14), cancer outcomes (15–18), neuropsychological performance (19–21) and mental health (22–25).

**Figure 1:**
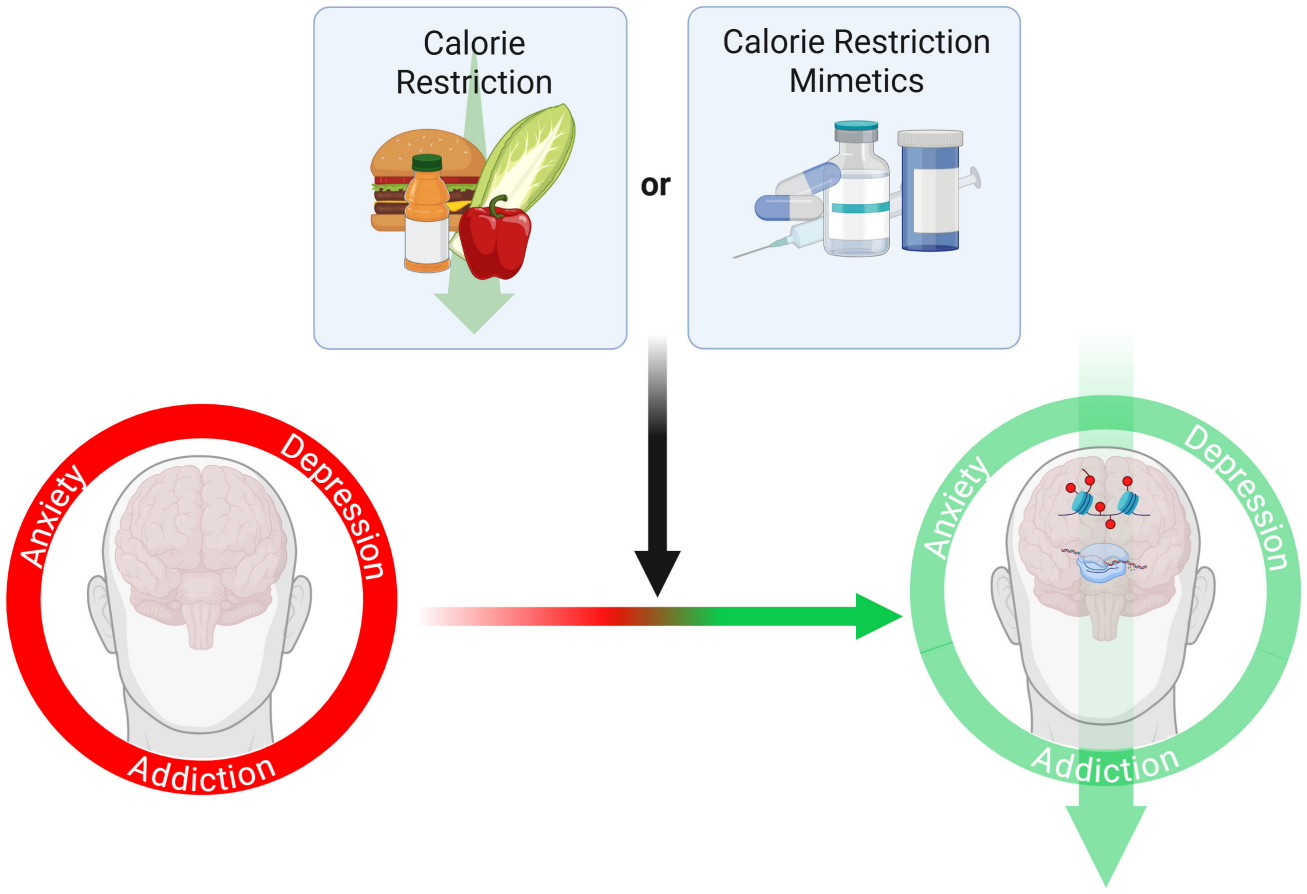
Understanding the genetic signatures induced by CR can lead to the development of novel treatments for mental health conditions, including anxiety disorders, mood disorders such as depression, and substance use disorders. Created in BioRender. Boskovic, Z. (2025) https://BioRender.com/epcts6e.

While there is mounting evidence for the benefits of CR across a variety of physiological and psychological outcomes, adherence to this regime is a key factor in determining the clinical utility of CR. Long-term CR trials often see declining compliance over time, though strategies like counseling, monitoring, and diet tracking can help improve it (26, 27). In the Comprehensive Assessment of the Long-term Effects of Reducing Intake of Energy (CALERIE) study, adherence to a 25% CR goal dropped significantly after 20 weeks (28). Clinical dropout rates vary widely, ranging from single digits to 40%. In one year-long study, dropout rates were 38% for alternate-day modified fasting, 29% for CR, and 26% for the control group (29). To this end, it has been proposed that CR mimetics (CRMs) - which can mimic one or more physiological, metabolic, and hormonal signaling cascades of CR - can be used to administer the positive health outcomes of CR without dietary restriction [**Figure 1**, (30, 31)]. While there have been significant advances in the development of CRMs, their potential in improving outcomes in individuals with mental health disorders is still under investigation (32–35).

Given the limitations of existing psychiatric treatments, there is a critical need to explore novel, mechanism-based approaches that address the underlying biological drivers of mental illness. In this review, we summarize emerging evidence that suggests that CR, and in particular CRMs, may offer such an approach with promising effects on mental health in preclinical and clinical settings. We will explore the molecular mechanisms that may mediate their effects and examine the therapeutic potential of CRMs as more sustainable alternatives to current treatments and to CR alone. We also highlight emerging molecular targets that could be leveraged for the development and screening of next-generation CRMs, offering a new avenue for innovation in psychiatric therapeutics.

## 2 Calorie restriction and its effect on anxiety and depression

Anxiety and depression are highly comorbid, and a strong predictor of major depressive disorder is comorbid anxiety disorder (36, 37). Common therapeutic approaches have included psychotherapy and psychopharmacology. However, anxiolytics and antidepressants are not always effective and can lead to drug abuse, failure to respond, lag time for therapeutic effect, dependence, and/or intolerance (6, 7). As such, the use of CR as a non-pharmacological tool to assist in the treatment of neuropsychiatric disorders has the potential to have a profound impact on the treatment of these debilitating conditions. There is a growing body of evidence to support this line of reasoning, and while traditionally most of this has come from pre-clinical studies discussed in the following section, we will also present the growing clinical data supporting CR’s therapeutic role and its limitations.

### 2.1 Preclinical evidence on therapeutic effects of calorie restriction

The effects of CR on anxiety-like behavior in animal models vary, dependent on length, severity, and age at initiation of CR. When young rats (about 4 weeks old) were put on a severe diet that cut their calories by 50% for five weeks, they showed increased signs of anxiety (38). In contrast, studies in adult rats have found that a more moderate reduction - about 25% fewer calories - can reduce anxiety, especially when continued for several weeks or even up to 18 months. These effects were observed using common behavioral tests like the elevated plus maze and open field tests (39–42). The results in older rats are more mixed: a short-term CR (3 months) starting at 21 months old increased anxiety, but a longer CR (6 months) starting at the same age reduced it (43). Interestingly, starting CR a bit earlier at 18 months also reduced anxiety levels. These findings suggest that CR can help reduce anxiety even in older animals, but the length and timing of the intervention matter. Overall, mild CR in fully grown animals seems to have the most consistent anxiolytic effect.

Researchers have also explored whether CR can reduce depression-like behaviors in animal models. In the widely used forced swim test, which measures behavioral despair, mice on a CR diet showed less immobility - suggesting a potential antidepressant-like effect (44, 45). Another way to assess depression-related behavior is by examining social withdrawal. Mice on a 25% CR diet showed increased social interaction compared to controls, indicating reduced social withdrawal (46). Notably, even short-term CR (10 days at 40%) reversed social deficits caused by social stress, an effect linked to changes in orexin signaling - a brain system involved in appetite and emotional regulation (47). In aggressive behavior tests, CR mice were more sociable and less aggressive, effects that were lost in mice lacking the cAMP Response Element-binding (CREB) protein, suggesting that CREB is necessary for CR’s behavioral benefits (48). Other findings point to astrocytes as possible mediators of CR’s antidepressant effects. CR failed to reduce depression-like behavior in mice lacking IP3R2, a receptor involved in calcium signaling in astrocytes, suggesting this pathway plays a key role (45).

While encouraging, the observed findings come with several important limitations. In animal models, the effects of CR are highly nuanced and depend on variables such as the age at intervention, the severity and duration of restriction, and the specific behavioral assays used. Additionally, differences across species and sex further complicate the translation of these results to human populations. Despite these challenges, preclinical studies offer valuable insight into potential mechanisms by which CR may exert therapeutic effects on psychiatric symptoms. Beyond the highlighted molecular pathways like CREB and orexin signaling, CR also modulates key neurotransmitter systems implicated in mood regulation, including dopamine (43, 49, 50), serotonin (51, 52), and norepinephrine (53, 54). These findings suggest that CR may influence anxiety and depression through a complex interplay of neuromodulation, intracellular signaling cascades, and astrocyte function. Taken together, this body of evidence highlights promising molecular targets for future therapeutic development in psychiatry.

### 2.2 Clinical evidence on therapeutic effects of calorie restriction

While preclinical studies have long suggested that CR may alleviate behaviors associated with psychiatric disorders, human data supporting its use in clinical settings is only beginning to emerge. It is well-established that weight loss from interventions such as exercise, pharmacotherapy, or bariatric surgery can improve symptoms of anxiety and depression (55–59). However, it remains unclear whether these benefits stem from weight loss itself or mechanisms specific to CR. Human trials examining CR’s psychiatric effects are limited, often constrained by short durations (6–12 weeks), use of very low-calorie diets (VLCDs <800 kcal/day), and concerns about feasibility, adverse outcomes, and adherence (55, 58, 60, 61).

Nonetheless, human studies are encouraging with two recent meta-analyses reporting that CR in overweight and obese individuals reduced depressive symptoms (58, 59). Echoing findings from the preclinical space, human studies vary in duration of CR and its therapeutic benefits. While one study reports 30% CR over six months improved both anxiety and depression in obese participants (62), a recent randomized clinical trial indicated that improvements in anxiety and depression symptoms are observed as quickly as 15 days after initiation of CR in overweight and obese individuals and persisted for at least 60 days (61). However, the Clinical Study of Obesity and Intestinal Microbiota (ECOMI) trial showed that short-term CR (12 weeks) can lead to significant improvements in anxiety and depression scores in obese individuals only when combined with probiotic supplementation (63). Interestingly, a 2019 study undertaken in Spain showed that in obese individuals, therapeutic effects of CR on anxiety were only observed in women, while improvements in depression scores were observed in all participants (23). Encouragingly, human studies mimic preclinical findings demonstrating that CR can have significant effects on dopaminergic and serotonergic signaling in obese individuals (62, 64), providing mechanistic evidence for the therapeutic avenues of CR in humans.

While the aforementioned results are encouraging, they still do not fully differentiate whether the observed therapeutic benefits are due to CR, weight loss or a combination of both. To this end, the findings of the Comprehensive Assessment of the Long-term Effects of Reducing Intake of Energy (CALERIE) phase 1 (CALERIE-1) and phase 2 (CALERIE-2) study are particularly illuminating. CALERIE-1 was designed to emphasize adherence to a 25% CR over an extended period of time with no specific macronutrient composition (other than nutritional adequacy) being recommended (65). CALERIE-2 was a continuation of this study, with the aim to assess the beneficial effects of CR when participant’s weights stabilized following the initial weight loss (60). The findings of the latter study show individuals who undergo 25% CR for 2 years have significant improvements in mood and reported fewer mood disturbances (measured by Beck Depression Inventory - II). The authors indicate that this regime is feasible, has positive metabolic effects, and can improve mood with no negative impacts on health-related quality of life. These findings provide significant evidence that CR can mediate positive therapeutic outcomes on anxiety and depression independent of weight loss, suggesting that the positive observations in clinical trials performed in obese individuals are mediated in part or completely by CR-induced changes.

The preclinical and clinical findings outlined in this section are highly relevant to psychiatric practice, especially when considering adjunctive strategies for treatment-resistant depression, anxiety disorders, or comorbid obesity and mood dysfunction. While CR is not yet a mainstream therapeutic tool in psychiatry, its mechanistic overlap with known pathways involved in anxiety and depression -neurotransmitter modulation, CREB and orexin signaling, and astrocyte function - makes it a promising area for future translational research and clinical trials.

## 3 Calorie restriction and its role in treating addiction

Substance use disorders, including alcohol and drug addiction, remain a major global health challenge, contributing significantly to premature death and disability (66, 67). In 2021, it was estimated that 39.5 million people globally suffer from substance use disorder, of which only 20% received drug treatment (68). An estimated 400 million people suffer from alcohol use disorder, which was responsible for 6.7% of all premature mortality in 2019 (69). Although the role of CR in treating addiction in humans remains largely unexplored, animal studies offer intriguing insights. In the alcohol-preferring rat model, 25% CR reduced alcohol self-administration and suppressed relapse behaviors such as alcohol deprivation effects and cue-induced reinstatement of alcohol seeking (70, 71). These findings suggest that CR may reduce compulsive alcohol consumption and protect against relapse, key therapeutic goals in addiction treatment.

However, the protective benefits of CR can be complex, as other preclinical studies have shown that food-restricted animals tend, paradoxically, to increase their consumption of addictive substances. This may be a result of CR enhancing the rewarding properties of drugs of abuse and amplifying drug-seeking behavior (72–74). These processes seem to be mediated by stress-related neurocircuits, such as corticotropin-releasing factor (CRF) and dopamine transmission, but not corticosterone (75, 76). Both CRF and corticosterone are key mediators of behavioral, autonomic, and endocrine responses to stress, but only CRF, and not corticosterone, has been shown to contribute to relapse to heroin-seeking induced by stressors (77). These findings should be interpreted with caution as the CR regimen was short-term and relatively mild (15-20%), which begs the question whether longer or more severe CR might produce potentially beneficial effects on addiction-related behavior.

Emerging preclinical evidence suggests that chronic CR may have protective effects against addiction and relapse, particularly in alcohol use models, by modulating key neurobiological systems involved in reward and stress. While acute food restriction may increase vulnerability to drug-seeking behaviors, sustained, moderate CR could represent a promising, non-pharmacological strategy for managing substance use disorders. However, differences in physiology, psychological context, and environmental complexity between humans and preclinical models highlight significant translational limitations. Further research, including clinical studies to explore CR’s potential as a novel adjunctive treatment for addiction in psychiatric settings, is needed to determine the efficacy of CR-based interventions in patients with substance use disorders.

## 4 Calorie restriction mimetic candidates

Given the negative effects that the global obesity epidemic has on health and the economy, it comes as no surprise that pharmaceutical compounds that mimic the metabolic effects of CR for the purposes of weight loss have gained attention from the public and scientific community (78). The impact these drugs have had in the clinical treatment of obesity is evidenced by the fact that the journal *Science* named GLP-1 drugs the breakthrough of 2023 (79). Coupled with the practical limitation of human subjects adhering to CR in non-controlled environments, we have seen increased research into CRMs - which can mimic one or more physiological, metabolic, and hormonal signaling cascades of CR without dietary restrictions. In the following section, we focus on the role that some of these compounds may have in the treatment of mental health disorders.

### 4.1 Resveratrol

Resveratrol, a natural polyphenol found in grapes, exhibits a wide range of cellular effects, including antioxidant, anti-inflammatory, anti-carcinogenic, neuroprotective, and anti-aging properties (80, 81). Resveratrol is believed to stimulate the activity of sirtuin 1 (SIRT1) and phosphorylated-5’adenosine monophosphate-activated protein kinase (AMPK) in multiple tissues, yet its exact mechanism of action is elusive because SIRT1 and AMPK can regulate each other and share many common target molecules (82, 83).

In relation to anxiety and depression, preclinical studies have shown that resveratrol appears to have antidepressant and anxiolytic effects, possibly by inhibiting phosphodiesterase-4 (PDE4) - an enzyme that breaks down cAMP, a molecule involved in neuroplasticity and mood regulation (84). Through PDE4 inhibition, resveratrol may indirectly enhance signaling pathways such as PKA-CREB-BDNF, which are frequently dysregulated in mood disorders. The effect of resveratrol on this pathway is of particular interest as PDE4 inhibition has been studied for some time as a potential anxiolytic and antidepressant pathway (85–88). Over the past 10 years, numerous PDE4 inhibitors have been discovered (89, 90) and a number of these have entered clinical trials (91). While most trials have focused on treating non-psychiatric conditions, such as rheumatoid arthritis, chronic obstructive pulmonary disease (COPD), and asthma, some of these compounds have been trialed for their effects on anxiety and depressive disorders. The earliest and most investigated PDE4 inhibitor, Rolipram, was initially trialed with no success (92), but has since been brought back into Phase I with results yet to be reported (91). Given that Rolipram is a first-generation PDE4 inhibitor, efforts have been made to improve potency, selectivity, and improve side effect profiles. This led to the development of numerous novel inhibitors in clinical trials for the treatment of depressive and anxiety disorders. These include Roflumilast (Phase I), Zatomilast (Phase II) and GSK356278 (Phase I), with the trials either still in progress or results to be submitted (91). Beyond PDE4 inhibition, some evidence does suggest resveratrol may positively affect mood, by modulating key neurotransmitter systems including dopamine, serotonin, and NPY (93), influencing BDNF-mediated neuroplasticity (94) or reducing systemic inflammation (95).

There is also growing interest in resveratrol’s potential to treat substance use disorders. In animal models, resveratrol impaired the acquisition of drug-associated behaviors and prevented relapse in alcohol-conditioned place preference paradigms (96). Notably, its efficacy was comparable to acamprosate, a clinically used drug for managing alcohol dependence. Similar effects were observed with heroin, where resveratrol reduced addiction-like behaviors (97, 98), suggesting it may modulate common reward pathways or stress-related circuits involved in addictive behavior.

Resveratrol shows promise in preclinical studies for improving mood, reducing anxiety, and decreasing addiction-like behaviors, likely through mechanisms involving PDE4 inhibition, enhanced cAMP signaling, modulation of key neurotransmitters, and activation of neuroprotective- and neuroplasticity-related pathways like SIRT1 and BDNF. However, its clinical utility remains uncertain due to limited and inconclusive human data. Furthermore, while there is overlap between the effects of resveratrol and CR, resveratrol does not mimic all aspects of CR (99) and widespread use of resveratrol has been hindered by its low bioavailability, though improvements have been made by manipulating its formulation (81, 100).

### 4.2 Rapamycin

Rapamycin is an inhibitor of the mechanistic target of rapamycin (mTOR) protein kinase pathway and has been long investigated as a CRM candidate. mTOR is a key component of a signaling network that can sense local (e.g. glucose, oxygen, amino acids) and systemic (e.g. insulin and insulin-like growth factor-1) nutrient status and respond accordingly to regulate cell growth, metabolism, proliferation, and survival (101). There have been reports that rapamycin may increase anxiety-like behavior (102, 103), however, considering the strength of evidence that has been published so far, rapamycin can ameliorate depressive- and anxiety-like behavior induced by a variety of conditions (104–106).

These effects appear to be mediated by several interconnected molecular pathways. One key mechanism is enhanced autophagy, a cellular process involved in the degradation and recycling of damaged cellular components (107). Chronic stress and depression are associated with impaired autophagic activity in the brain, particularly in regions such as the hippocampus and prefrontal cortex (108). Rapamycin restores this deficit by inhibiting mTORC1, thereby activating autophagy, which contributes to cellular homeostasis and neuronal resilience (105). Restored autophagic flux may help clear dysfunctional mitochondria, reduce oxidative stress, and promote neuroplasticity - all of which are implicated in the pathophysiology of depression.

Additionally, rapamycin has been shown to increase levels of BDNF (109), a critical neurotrophin involved in synaptic plasticity, neuronal survival, and cognitive function. Decreased BDNF levels are consistently observed in individuals with depression, and enhancing BDNF signaling is a known mechanism of action for many antidepressants (110). By increasing BDNF expression, rapamycin may support neuronal growth and synaptic connectivity, helping to reverse the structural and functional brain changes associated with mood disorders (105).

Another mechanism includes enhanced myelination, particularly in the medial prefrontal cortex, a region highly relevant to emotional regulation. Increased myelination may improve the speed and efficiency of neuronal signaling, thereby supporting improved behavioral responses to stress and emotional stimuli (106).

Rapamycin can also reduce motivational responding to cocaine using the progressive ratio testing after animals were trained for self-administration (111), and reduced cocaine-related relapse behavior (112). These preclinical results are corroborated by results from a double-blind clinical trial in abstinent heroin addicts, which showed that a single dose of rapamycin can reduce heroin craving when exposed to drug-related imagery (113). This suggests that there may also be a therapeutic role for rapamycin in the treatment of substance use disorders.

Although rapamycin can be considered a CRM, there are differences in mechanisms when compared to CR (114–116). Liver transcriptome and metabolome analysis in mice showed that approximately 80% of the transcripts were distinct to either CR or rapamycin treatment, with 20% overlapping (115). This distinct molecular signature suggests these interventions might have complementary effects, though further research is needed to determine if these effects are additive in the liver, and whether similar patterns exist in other tissues. There are apprehensions in taking rapamycin for extended time periods due to the lack of long-term studies and underassessment of rapamycin’s effects on the respiratory, digestive, renal, and reproductive systems (117). However, a recent meta-analysis showed that rapamycin (and its derivatives) can be considered tolerable in humans, as no serious adverse events have been associated with its use (117).

While rapamycin shows promise as a CRM, and the collective neural pathways affected by rapamycin position it as an intriguing candidate for novel psychiatric treatment, further translational research is needed to evaluate its clinical viability. While direct clinical trials of rapamycin for treating depression and anxiety are limited, studies such as the effects of combination therapy with ketamine on alleviating depressive symptoms (118) may provide critical insight into the therapeutic effect of rapamycin in the human population and pave the way for future clinical studies.

### 4.3 Semaglutide: a CRM-adjacent therapeutic

Semaglutide was developed initially to treat type 2 diabetes mellitus (T2DM) and is an analog to GLP-1 that has significant effects on glycemic control and body weight regulation by binding to GLP-1 receptors. Being widely distributed in the brain, GLP-1 signaling has many functions, with activation of GLP-1 receptors in the hypothalamus, brain stem, and septal nucleus believed to mediate the effect of semaglutide on appetite and body weight regulation (119).

While semaglutide is not traditionally classified as a CRM, we include it in this review for three key reasons. Firstly, GLP-1 receptor agonists such as semaglutide engage signaling pathways that overlap with those activated by CR, including pathways related to energy metabolism, inflammation, and cellular stress resistance (120). Exploring these shared mechanisms may offer valuable insight into how CR mediates its neuropsychiatric benefits. Secondly, although some of semaglutide’s mental health benefits may stem from weight loss (121), the associated reduction in caloric intake (122) could itself activate neuromolecular circuits similar to those triggered by CR, including modulation of reward processing, neuroplasticity, and stress resilience. Finally, given the rapid and widespread growth in both preclinical and clinical research on semaglutide - particularly regarding its impact on mood, cognition, and addictive behaviors discussed below - it presents a timely and relevant opportunity to evaluate how metabolic interventions may influence psychiatric outcomes. Excluding such a widely studied compound from the CRM discussion would miss a critical perspective on the evolving landscape investigating the interface between metabolism and mental health.

Beyond its metabolic effects, semaglutide engages central pathways relevant to psychiatric function, further supporting its inclusion as a CRM-adjacent compound. Activation of GLP-1 receptors in the amygdala has been linked to increased dopamine turnover and reduced food reward, indicating semaglutide’s influence on reward processing and motivational behavior (123). This dopaminergic modulation may contribute to the drug’s effects on learning, memory, and emotional regulation. Supporting this, GLP-1 receptor knockout mice exhibit deficits in memory and synaptic plasticity, including impaired long-term potentiation - an essential mechanism for learning and mood stability (124). Furthermore, GLP-1 peptide seems to alleviate depressive symptoms through several mechanisms shared by CR, including neuroinflammation, neurotransmitter modulation and synaptic function (125). Preclinical studies have shown that semaglutide improves cognitive performance and reduces anxiety- and depression-like behaviors in mice exposed to a high-fat diet, suggesting neuroprotective and mood-stabilizing properties (126). Importantly, semaglutide has also demonstrated promise in addiction models. It reduces alcohol intake in a dose-dependent manner in binge-drinking paradigms (127) and prevents relapse-like behavior following alcohol withdrawal in both male and female rats (128). Semaglutide also seems to be faster-acting than its predecessor, liraglutide, for the treatment of alcohol use disorder (129), pointing to its potential utility in substance use disorders.

While limited, emerging clinical data demonstrates that semaglutide can have beneficial effects on addiction and addiction-like behaviors. In a cross-sectional study of 69 obese patients, it was shown that semaglutide was effective in ameliorating emotional eating and other abnormal eating patterns (130), while in a retrospective cohort study the beneficial therapeutic effect was expanded to include reduction of binge eating behavior (131). In the space of alcohol-use disorder (AUD), obese individuals taking semaglutide self-reported less alcohol consumption and fewer binge drinking episodes in comparison to controls and to before semaglutide treatment (132). A more recent trial in non-obese patients with AUD (133) showed that even in low doses semaglutide significantly reduced alcohol intake and cravings. The efficacy of this treatment for AUD is still expanding, with two currently ongoing trials (Semaglutide Therapy for Alcohol Reduction - Tulsa (STAR-T) NCT05891587; Clinical Trial of Rybelsus (Semaglutide) Among Adults With Alcohol Use Disorder NCT05892432) investigating the effects of low-dose treatment for a period of 12 weeks, and a higher dose treatment for a period of 8 weeks, respectively. Taken together, the encouraging findings on the use of this compound for the treatment of addictive disorders further emphasize the immense potential of metabolic interventions in the field of psychiatry.

Despite encouraging clinical data showing the effectiveness of semaglutide for the treatment of addictive behaviors, there have been concerns that semaglutide use is associated with an increased number of suicidal ideations compared to other antidiabetic medications (134) and has adverse effects on depression (135). However, a recent retrospective cohort study, which included 240,618 overweight or obese patients who were prescribed either semaglutide, or non-GLP1R-agonist anti-obesity medications, did not find increased risk of suicidal ideation with semaglutide use (136). This was corroborated by another retrospective study that found that instead, after multiple testing correction, semaglutide was associated with reduced risk for several such outcomes (137).

Although semaglutide shows much promise as a glucose and weight management pharmacological tool, if used for some of the aforementioned benefits outside of weight loss, there would be concerns regarding its long-term safety profile and in patients where weight loss is not desirable. There are also concerns about long-term benefits since upon cessation of semaglutide treatment, patients tend to regain a significant portion of the lost weight (138, 139), with reports that patients who underwent 68 weeks of treatment with semaglutide rapidly regained two-thirds of previously lost weight, and cardiometabolic measures reverted to baseline after 120 weeks following semaglutide withdrawal (139). It stands to reason that any beneficial effects of semaglutide outside of weight loss would follow similar trajectories once treatment has been discontinued. To fully understand whether this is the case, there is a need for follow-up and longitudinal clinical studies specifically aimed at assessing long-term effects of semaglutide on psychiatric conditions.

With limited but rapidly expanding clinical data assessing the efficacy of semaglutide for the treatment of psychiatric disorders (140), caution should be taken when interpreting and translating preclinical findings. Nevertheless, the positive impact of this compound on T2DM management has been profound, with preclinical and limited clinical observations on the beneficial effects of semaglutide in anxiety, depression, and addiction further highlighting the significant potential of utilizing this compound as a mental health treatment.

## 5 Development of next-generation CRMs

The current understanding of mechanisms by which CR exerts its effects spans multiple neurophysiological processes, including neurogenesis, synaptic plasticity, neurotransmitter modulation, oxidative stress, nutrient-sensing, autophagy, and inflammatory pathways (141). Although these pathways can regulate CR’s effects independently, there is significant overlap between their signaling cascades. We have so far reviewed evidence of a variety of beneficial effects of CR and CRMs on mental health disorders. However, we suggest that to maximize therapeutic benefits, future CRMs need to target a very specific aspect of the CR signaling pathway in order to avoid some unwanted effects within the target population.

While we have explored several mechanisms by which CR could influence mental health, there remains vast potential for expanding the pathways of next-generation CRMs. In the following section, we highlight the cAMP/protein kinase A (PKA)/CREB cascade and specific epigenetic pathways, as we believe they have potential to be leveraged for development into innovative CRM treatments for mental health disorders.

### 5.1 cAMP/PKA/CREB signaling pathway

The cAMP/PKA/CREB signaling pathway, also known as the cAMP-dependent pathway, has a role in regulating neuronal growth and development, synaptic plasticity, neurogenesis and memory consolidation (48, 142). The canonical cAMP/PKA/CREB pathway for gene transcription lies downstream of ligand binding to an appropriate G-protein coupled receptor, causing cAMP production, that in turn activates PKA to directly phosphorylate CREB resulting in histone modification and increased transcription (143, 144). The CR signaling pathway has significant overlap with the cAMP/PKA/CREB cascade with both CR and exogenous administration of cAMP in mice increasing SIRT1 mRNA expression to prevent oxidative stress, increase longevity and improve neuropsychological functioning (145).

The most likely candidate within this pathway through which CR mediates its mental health benefits is CREB, as CREB-deficient animals show limited neurophysiological improvements in response to CR (48). CREB is also a mediator of experience-based neuroadaptations and regulator of the addictive qualities of drugs of abuse (146) and its expression in the hippocampus is directly correlated with antidepressant effects (147–149). As highlighted in section 4.1 above, numerous novel therapies targeting CREB through PDE4 inhibition have been developed and are being trialed as treatments for mental health disorders, further emphasizing CREB signaling as a promising therapeutic target.

However, therapeutic targeting of CREB through direct or indirect pharmacological intervention remains challenging due to its role in the ubiquitous cAMP signaling cascade. This cascade, which involves cAMP, PDE4, PKA, and CREB, mediates, modulates, and regulates diverse cellular functions throughout both central and peripheral systems, making therapeutic effects difficult to isolate and predict. Further exploration in the overlap between CR and the CREB pathway may reveal targets that could be modulated to specifically exert beneficial effects on mental health without having adverse outcomes on other neuronal functions.

### 5.2 Epigenetics

A strong implication stemming from the previously reported improvement of neuropsychological function in the offspring of CR rodents is that such an intervention causes inheritable epigenetic modifications (150). CR induces epigenetic changes mainly via DNA methylation [reviewed in (151)], histone modification, and non-coding RNA regulation – processes that have only been partially investigated in the context of CR (152). In particular, CR has been found to affect the DNA methylation pattern of genes involved in metabolism, oxidative stress and aging (153–155) in different tissue types such as in the liver (156), kidney (157) and brain (158, 159). In mice, CR causes hypermethylation of methyltransferase promoters (155) and may have histone modification abilities due to its ability to upregulate SIRT which has histone deacetylase properties (160). Histone deacetylase activity has been also observed to be increased by CR and was suggested to facilitate CR’s ability to act as a protective mechanism (161). There has been limited evidence of changes in non-coding RNA by CR in mice brains, however, researchers have found that 40% CR for 2 years (162) and 15% CR for 72 weeks (163) can modulate several microRNAs involved in apoptosis, neuroprotection, neurogenesis, neuronal survival, axon guidance and histone regulation (164).

CR’s potential as a treatment for mental health disorders, combined with its ability to induce epigenetic modifications, is particularly relevant given that aberrant epigenetic changes contribute to the pathophysiology of mental health disorders and addiction (165–168). Epigenetic modification, predominately via gene-specific and genome-wide DNA methylome investigations, has been suggested to represent the physiological mechanistic link between environmental factors that can increase susceptibility to mental health disorders (169, 170). Abnormal changes in histone modification and microRNA deregulation have also been linked to mental health disorders (171–173). For example, blood of patients with MDD had lower levels of H3K4me3 levels, a marker for chromatin activation, in the promoter regions of *TNFAIP3. TLR4, TNIP2, miR-146a* and *miR-155* (172). The same study also showed that the severity of depression symptoms was associated with H3K4me3 levels at *TLR4* and *TNIP2* (172). In mouse models of anxiety, increases of histone deacetylation expression are observed and once inhibited, anxiety- and depressive-like symptoms are reversed (174, 175). As such, targeting epigenetic modifications caused by CR may be a promising avenue for novel CRM development.

## 6 Conclusions

In this review, we have examined the growing body of evidence supporting the potential role of CR and CRMs in the treatment of key psychiatric disorders, including anxiety, depression, and addiction. While CR has long been associated with increased lifespan and improved metabolic health, accumulating preclinical findings suggest that it can have beneficial impacts on mood and behavior. Specifically, CR exerts mild but consistent anxiolytic effects when initiated during young adulthood, and shows promise in alleviating depression-associated behaviors, such as social withdrawal, aggression, and modest improvements in the forced swim test. These effects seem to be achieved through modulation of key mood-related pathways, including serotonin, dopamine, and orexin signaling, CREB activity, and astrocyte function. In the case of addiction, in particular alcohol use disorder, CR may play an adjunctive role by reducing relapse risk through modulation of neurobiological systems involved in reward and stress, a finding largely restricted to the preclinical context.

A critical limitation of preclinical findings is that the efficacy of CR is not uniform - it is highly dependent on the timing, duration, and degree of caloric restriction. The most favorable outcomes are observed with moderate restriction (20–40%) maintained for extended periods (typically two months or longer) and initiated in early adulthood. In contrast, CR introduced later in life or under more severe regimens can be ineffective or even counterproductive. However, translation from preclinical models to psychiatric practice remains limited.

CR modulates a wide array of signaling pathways - such as the cAMP/PKA/CREB axis, orexin signaling, and epigenetic regulators - yet a more nuanced understanding of how these mechanisms converge to affect behavior in humans is urgently needed. While the majority of the reviewed clinical findings report positive effects of CR on mood disorders, they were performed in overweight and obese populations, confounding interpretation. Namely, it is difficult to discern whether the positive effects come from CR, weight loss, or a combination of both. However, the findings of the CALERIE study suggest that CR alone could be sufficient to provide significant mood improvements. Nonetheless, weight loss associated with CR may not be clinically appropriate in all populations, such as those with cancer or eating disorders, limiting its utility as a broadly applicable intervention.

These limitations underscore the clinical potential of CRMs - compounds that mimic the molecular effects of CR without dietary restriction. Among the compounds discussed in this review - resveratrol, rapamycin, and semaglutide - all show mechanistic and behavioral evidence of efficacy in models of anxiety, depression, and addiction. This is further evidenced by the increased research of these compounds in the clinical setting. Importantly, these compounds may offer more targeted therapeutic benefits than CR itself due to their action on specific molecular pathways. For instance, resveratrol’s inhibition of PDE4 leads to enhanced cAMP signaling; rapamycin promotes autophagy and elevates BDNF levels; and semaglutide, a GLP-1 receptor agonist, influences reward-related behaviors and reduces substance-seeking in preclinical models through neurotransmitter modulation such as dopamine.

Future research should focus on several fronts:

- Clinical Trials – More rigorously designed trials are needed to directly test CR and CRM efficacy in patients with anxiety, depression, and substance use disorders. In particular, further clinical studies on the effectiveness of metabolic interventions in non-obese populations are needed to understand the potency of this class of therapeutics.
- Mechanistic Studies – Clarifying the molecular cascades that link metabolic changes to neuropsychiatric outcomes will be critical. Emphasis should be given to pathways involving neurotrophic factors, the cAMP signaling pathway, critical neurotransmitter systems, and key epigenetic modifications.
- Personalized Interventions – Identifying patient-specific biomarkers or psychiatric subtypes that are most likely to benefit from CR or CRM-based therapies could maximize therapeutic success while minimizing risk.
- Novel CRMs – While CR and CRMs affect numerous molecular pathways, many of their modulatory mechanisms remain to be fully elucidated. This underscores the urgent need for the development of novel compounds that may provide all the benefits of CR with limited side effects.

In summary, the intersection of metabolism and mental health represents a novel and promising frontier in psychiatry. CR and CRMs offer a compelling therapeutic avenue by modulating core biological processes, one that warrants deeper investigation and clinical translation.
